# A Zinc Oxide Nanorod Ammonia Microsensor Integrated with a Readout Circuit on-a-Chip

**DOI:** 10.3390/s111211112

**Published:** 2011-11-28

**Authors:** Ming-Zhi Yang, Ching-Liang Dai, Chyan-Chyi Wu

**Affiliations:** 1 Department of Mechanical Engineering, National Chung Hsing University, Taichung 402, Taiwan; E-Mail: d099061005@mail.nchu.edu.tw; 2 Department of Mechanical and Electro-Mechanical Engineering, Tamkang University, Tamsui 251, Taiwan; E-Mail: ccwu@mail.tku.edu.tw

**Keywords:** ammonia microsensor, zinc oxide film, nanorod, readout circuit

## Abstract

A zinc oxide nanorod ammonia microsensor integrated with a readout circuit on-a-chip fabricated using the commercial 0.35 μm complementary metal oxide semiconductor (CMOS) process was investigated. The structure of the ammonia sensor is composed of a sensitive film and polysilicon electrodes. The ammonia sensor requires a post-process to etch the sacrificial layer, and to coat the sensitive film on the polysilicon electrodes. The sensitive film that is prepared by a hydrothermal method is made of zinc oxide. The sensor resistance changes when the sensitive film adsorbs or desorbs ammonia gas. The readout circuit is used to convert the sensor resistance into the voltage output. Experiments show that the ammonia sensor has a sensitivity of about 1.5 mV/ppm at room temperature.

## 1. Introduction

Ammonia sensors are important devices that can be applied in agriculture, biomedicine and industry. Recently, various microsensors have been fabricated using microelectromechanical system (MEMS) technology, and they offer the benefits of small size, low cost, high performance and easy mass-production [1]. Several researchers have employed MEMS technology to develop ammonia microsensors. For instance, Li and Li [2] used surface and bulk micromachining processes to make a micro gas sensor consisting of piezoresistive SiO_2_ cantilever beams. An ammonia sensitive film of 11-mercaaptoundecanoic acid was coated on the piezoresistive cantilever beams. The sensor was combined with a linear amplifier, and it had an output voltage of about 7 μV in 1 ppm NH_3_. Lee *et al.* [3] presented a resistive ammonia microsensor fabricated by bulk micromachining. The sensor comprised a SU-8 adhesion layer, an ammonia sensitive film and interdigitated Pt electrodes, where the ammonia sensitive film was polyaniline. The sensitivity of the ammonia sensor was about 40% at 50 ppm ammonia. Llobet *et al.* [4] proposed micro gas sensors manufactured by a screen-printing technique. The sensors were constructed by a polysilicon heating resistor, a sensitive layer, insulating layers and platinum electrodes, in which the sensitive layer was nanopowder tin oxide. The gas sensors were sensitive to ammonia vapor. Triantafyllopoulou *et al.* [5] utilized porous silicon techniques to produce ammonia microsensors. Two different nanostructured sensitive materials, SnO_2_/Pd and WO_3_/Cr, were deposited on the micro-hotplates in the sensors, and the SnO_2_/Pd sensor was more sensitive to ammonia. Briand *et al.* [6] employed anisotropic bulk silicon micromachining to fabricate a low-power consumption metal-oxide-semiconductor field-effect transistor (MOSFET) array gas sensor. The structure of the sensor contained a heating resistor, a temperature sensor and four MOSFETs located in a silicon island suspended by a dielectric membrane. The sensor was sensitive to ammonia and hydrogen. The ammonia sensors, proposed by Li *et al.* [2], Lee *et al.* [3], Llobet *et al.* [4], Triantafyllopoulou *et al.* [5], Briand *et al.* [6], were not integrated with circuitry on-a-chip. But package cost can be reduced and performances enhanced if microsensors are integrated with circuitry on-a-chip. In this work, an ammonia sensor integrated with a readout circuit-on-a-chip is developed.

Fabrication of MEMS devices using the commercial CMOS process is called the CMOS-MEMS technique [7–10]. Microdevices manufactured by this technique can be integrated with circuits as a system-on-a-chip (SOC) due to their compatibility with the CMOS process. In this study we utilize the CMOS-MEMS technique to develop an ammonia sensor with a readout circuit-on-a-chip. The sensitive film is zinc oxide prepared by the hydrothermal method. The sensor needs a post-process to coat the sensitive film. The post-process includes etching the sacrificial oxide layer and coating the sensitive film. The ammonia sensor produces a change in resistance as the sensitive film absorbs or desorbs ammonia, and the readout circuit converts the resistance variation of the sensor into the output voltage.

## 2. Structure of the Ammonia Sensor

The integrated sensor chip consists of an ammonia sensor and a readout circuit, and the chip area is about 1 mm^2^. The ammonia sensor is composed of a sensitive film and polysilicon electrodes. The sensitive film is coated on the polysilicon electrodes. The area of the sensitive film is about 400 × 640 μm^2^, and its thickness is about 10 μm. The ammonia sensor produces a change in resistance when the sensitive film adsorbs or desorbs ammonia. The sensor without heater works at room temperature. The readout circuit is utilized to convert the resistance of the ammonia sensor into the voltage output.

Zinc oxide was adopted as the sensitive material for the ammonia sensor. The sensing mechanism of zinc oxide to ammonia has been reported [11]. Zinc oxide is an n-type semiconductor oxide material. At room temperature, Atmospheric oxygen molecules are absorbed on the surface of zinc oxide since they take electrons from the conduction band of ZnO, and the reaction is given by:

(1) $$\frac{1}{2}O_{2}(\text{gas})+{ne}^{-}\to {2O}_{\mathrm{ads}}^{n-}$$

where $O_{\mathrm{ads}}^{n-}$ is adsorbed oxygen (*n* = 0, 1, 2) and *e^−^* is electronic charge. When the zinc oxide is exposed to NH_3_ gas, the electrons trapped by the adsorptive states are released. The reactions can be expressed by [11]:

(2) $${2\mathrm{NH}}_{3}+{\left({7O}_{\mathrm{ads}}\right)}^{2-}\to {2\mathrm{NO}}_{2}+{3H}_{2}O+{14e}^{-}$$

(3) $${2\mathrm{NH}}_{3}+{3O}^{-}\to N_{2}+{3H}_{2}O+{3e}^{-}$$

According to Equations (2) and (3), the conductivity of zinc oxide changes upon the sensor absorbs ammonia gas.

**Figure 1.**
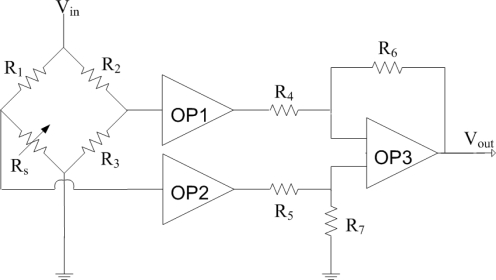
Readout circuit for the ammonia sensor.

**Figure 2.**
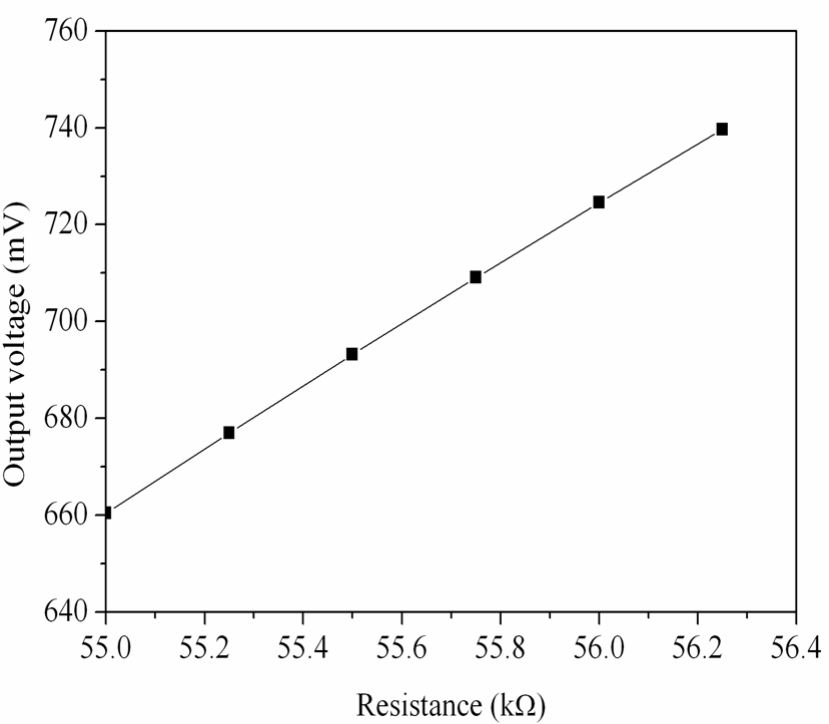
Simulated results of the output voltage for the ammonia sensor.

Figure 1 shows the readout circuit for the ammonia sensor [12], where *OP1*, *PO2* and *OP3* represent the operational amplifiers; *V_in_* is the input voltage of the circuit and *V_out_* is the output voltage of the circuit. The readout circuit is composed of a Wheatstone circuit, amplifiers and resistances. The Wheastone circuit comprises the resistance of the ammonia sensor (*R_s_*) and three resistances (*R_1_*, *R_2_* and *R_3_*). The resistance of the sensor, *R_s_*, generates variation as the sensitive film absorbs or desorbs ammonia. The resistance variation of the ammonia sensor uses the readout circuit to convert into the output voltage. This design used *R_1_* = 50 kΩ, *R_2_* = 50 kΩ, *R_3_* = 50 kΩ, *R_4_* = 10 kΩ, *R_5_* = 10 kΩ, *R_6_* = 15 kΩ and *R_7_* = 15 kΩ. The professional circuit simulation software, HSPICE, is utilized to simulate the output voltage of the readout circuit. Figure 2 presents the simulated results of output voltage for the readout circuit. In this simulation, the input voltage *V_in_* was 3 V, and the resistance of the sensor *R_s_* changed from 55 to 56.3 kΩ. The output voltage of the readout circuit varied from 660 to 740 mV as the resistance of the sensor changed from 55 to 56.3 kΩ.

## 3. Fabrication of the Ammonia Sensor

In the ammonia sensor, zinc oxide prepared by hydrothermal method was adopted as the ammonia sensitive material [13,14]. Preparation steps for the zinc oxide included: (1) zinc nitrate (Zn(NO_3_)_2_·6H_2_O, 2.9748 g) was dissolved in distilled water (50 mL) with vigorous stirring until a homogenous solution was formed; (2) sodium dodecyl sulfate (C_12_H_25_NaO_4_S, 0.144 g was added to the Zn(NO_3_)_2_·6H_2_O solution with stirring and cooled in ice water at 3 °C; (3) sodium hydroxide (NaOH, 0.48 g) was dissolved in distilled water (50 mL) with stirring; (4) the NaOH solution was added into the Zn(NO_3_)_2_·6H_2_O/C_12_H_25_NaO_4_S solution and stirred for 1 h at room temperature; (5) the mixing solution of Zn(NO_3_)_2_·6H_2_O/C_12_H_25_NaO_4_S/NaOH was transferred into a stainless steel autoclave sealed and maintained at 90 °C for 12 h; (6) the mixing solution was cooled to room temperature, and then the resulting product was filtered, rinsed with methanol and deionized water, and followed by dropping on the substrate using a precision micro-dropper. Finally, the film was calcined at 100 °C for 2 h.

**Figure 3.**
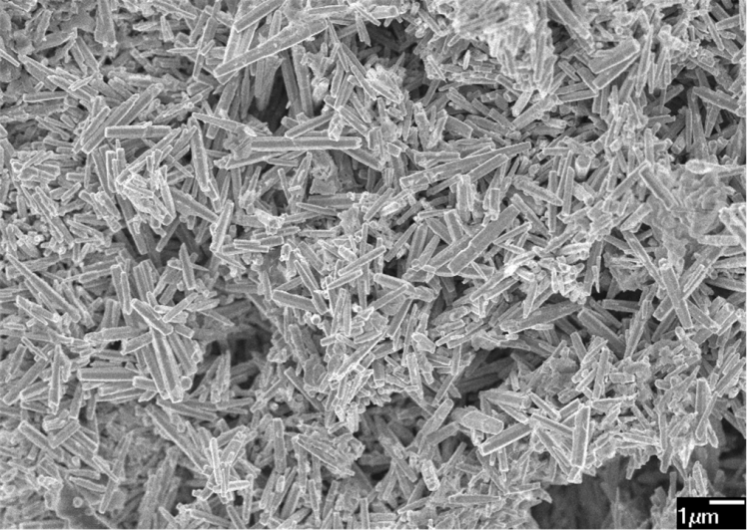
Scanning electron microscopy image of the zinc oxide film.

**Figure 4.**
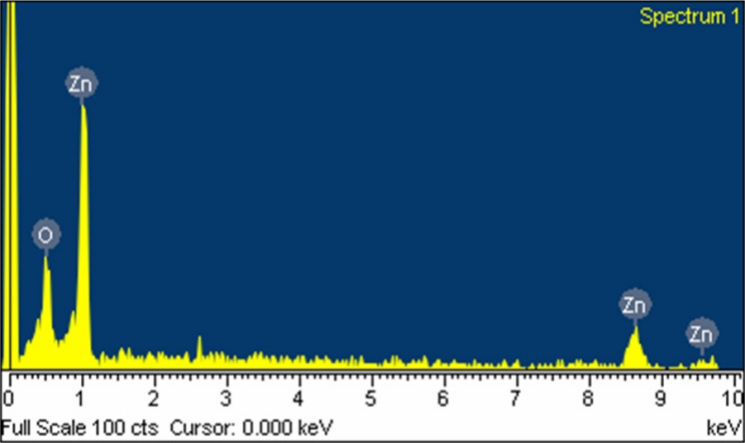
Elements of zinc oxide film measured by energy dispersive spectrometer.

The surface morphology of the zinc oxide film was measured by the scanning electron microscopy (JEOL JSM-6700F). Figure 3 shows a scanning electron microscopy image of the zinc oxide film. The sensitive film exhibits micro-porous and nanorod structures that helps to increase the sensing reaction since the film has porous structure. The pore density of the zinc oxide film was measured by an accelerated surface porosimetry analyzer. The results showed that the film had a BET (Brunauer emmett teller) surface volume of 8.5 m^2^/g and a total pore volume of 0.048 cm^2^/g. Elements of the zinc oxide film were detected by an energy dispersive spectrometer (Oxford INCA Energy 400). Figure 4 displays the measured results of the zinc oxide film by energy dispersive spectrometer. The main elements of the zinc oxide film were zinc and oxygen, and the film contained 22.98 wt% O and 77.02 wt% Zn.

**Figure 5.**
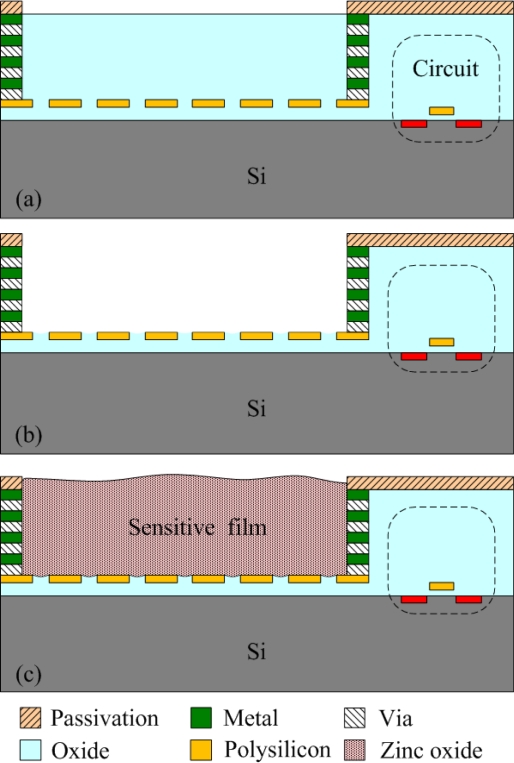
Fabrication process of the ammonia sensor: **(a)** after the CMOS process, **(b)** etching the sacrificial layer, **(c)** coating the sensing film.

**Figure 6.**
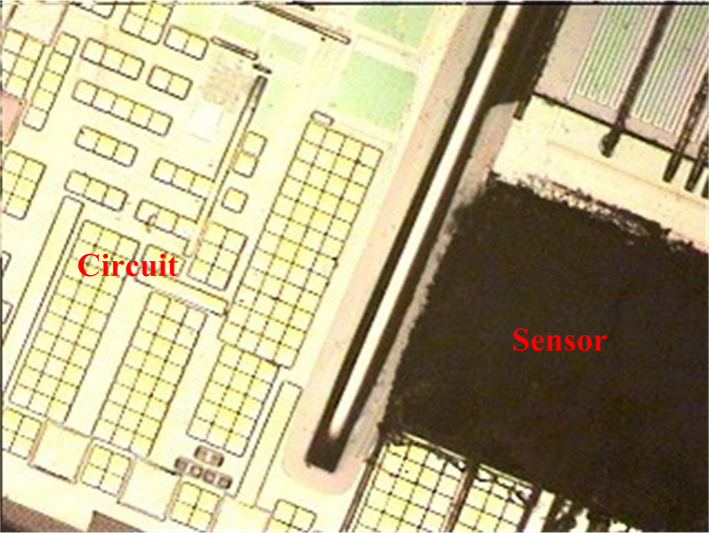
Image of the ammonia sensor after the post-process.

The commercial 0.35 μm CMOS process of the Taiwan Semiconductor Manufacturing Company (TSMC) was used to fabricate the integrated ammonia microsensor chip. After completion of the CMOS process, the ammonia sensor needed a post-process to etch the sacrificial layer and coat the sensitive film [15]. Figure 5 illustrates the fabrication flow of the ammonia microsensor. Figure 5(a) shows the cross-sectional view of the ammonia sensor after the CMOS processes. The polysilicon was used as the electrodes, and the silicon dioxide was adopted as the sacrificial layer. As shown in Figure 5(b), silox etchant [16] was employed to remove the sacrificial oxide layer and to expose the polysilicon electrodes. Figure 5(c) displays the sensitive film coated on the polysilicon electrodes. The zinc oxide slurry was dropped on the polysilicon electrodes using a precision micro-dropper, and the film was calcined at 100 °C for 2 h. Figure 6 depicts a photograph of the integrated ammonia microsensor after the post-process. The precision micro-dropper (MicroNami Inc., MKDR-303010) contains a micro-dropper, a CCD camera, a xyz step, and a controller. The zinc oxide slurry is putted into the micro-dropper. A coordinate position inputs to the controller, and the controller controls the micro-dropper moving to the chip position. The xyz step and the CCD camera are used to fine tuning the position between the micro-dropper and the sensing area on chip, and then a pneumatic pressure applies to the micro-dropper producing one drop slurry and dropping onto the sensing area. Each drop slurry amount is about 2.6 × 10^−6^ mL. Only one drop is used to fill the opening.

## 4. Results and Discussion

The performance of the ammonia sensor chip was measured using a test chamber, a power supply, an oscilloscope and an LCR meter. The humidity in the test chamber was maintained at 70%RH during testing.

**Figure 7.**
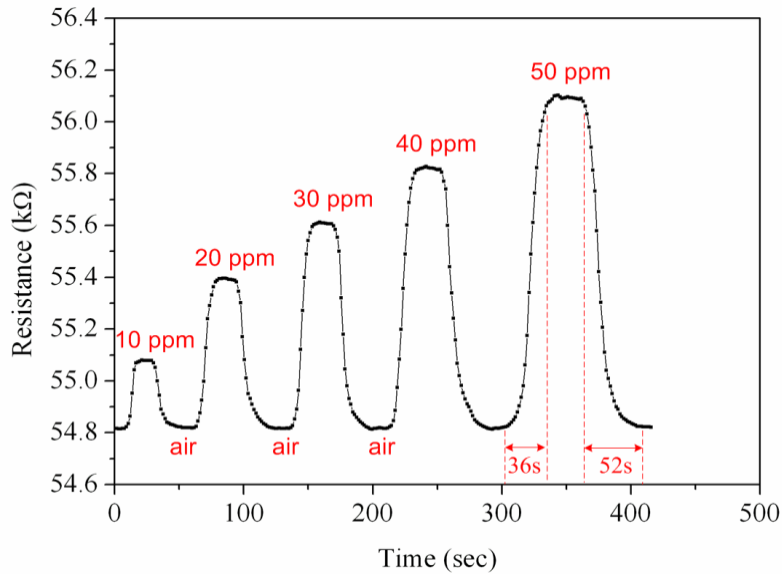
Test of the ammonia sensor.

**Figure 8.**
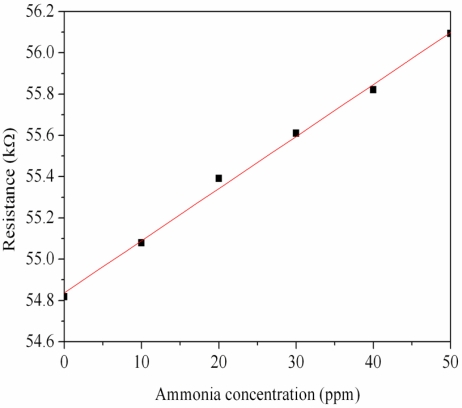
Measured results of the resistance for the ammonia sensors.

First, the ammonia sensor without readout circuit was tested in order to characterize the resistance variation of the sensor. The ammonia sensor chip without readout circuit was set in the test chamber, and its resistance variation under different ammonia concentrations was recorded by the LCR meter. Figure 7 demonstrates test of the ammonia sensor under different ammonia concentrations. The measured results revealed that the initial resistance of the ammonia sensor was about 54.82 kΩ (in air), and the resistance of the sensor increased to 56.09 kΩ at 50 ppm NH_3_. The ammonia sensor had a response time of about 36 sec at 50 ppm NH_3_ and a recovery time of 52 sec at 50 ppm NH_3_. Figure 8 shows the relation between resistance variation and ammonia concentration for the ammonia sensor. The resistance of the ammonia sensor increased as the concentration of ammonia increased.

**Figure 9.**
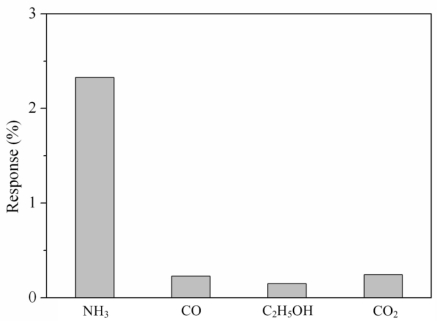
Response of the sensor to different gases.

Various gases included ammonia, carbon oxide, ethanol and carbon dioxide were each along provided to the tested chamber, and the LCR meter measured the resistance variation of the sensor. Figure 9 presents the response of the sensor under different gases at room temperature, where the response is defined by $\frac{\left|R-R_{0}\right|}{R_{0}}\times 100\%$; *R* is the measured resistance of the sensor with reaction gas and *R*_0_ is the original resistance of the sensor without reaction gas. In this investigation, ammonia, carbon oxide, ethanol and carbon dioxide concentrations were 50 ppm, 100 ppm, 100 ppm and 1,000 ppm, respectively, supplied to the tested chamber. As shown in Figure 9, the sensor had a response of 2.32% at 50 ppm NH_3_ and a response of 0.22% at 100 ppm CO. The results showed that the sensor was more sensitive to ammonia gas, and it was insensitive to carbon oxide, ethanol and carbon dioxide.

**Figure 10.**
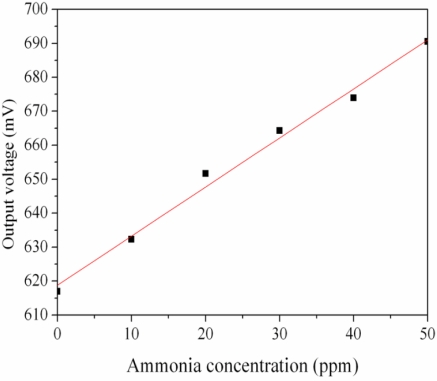
Measured results of the output voltage for the ammonia sensor.

The ammonia sensor with readout circuit was set in the test chamber and was measured under different ammonia concentrations at room temperature. The power supply provided a bias voltage of 3.3 V and an input voltage of 3 V to the readout circuit. The output voltage of the ammonia sensor was recorded by the oscilloscope. Figure 10 shows the relation between output voltage and ammonia concentration for the ammonia sensor. In this investigation, ammonia gas was provided from 1 to 50 ppm. The output voltage of the ammonia sensor varied from 617 to 691 mV as the concentration of ammonia gas changed from 1 to 50 ppm. The variation of the output voltage was 74 mV in 1–50 ppm NH_3_. Therefore, the integrated ammonia sensor had a sensitivity of about 1.5 mV/ppm when providing a bias voltage of 3.3 V and an input voltage of 3 V. The post-processing did not affect the function of the readout circuit and was compatible with the commercial CMOS process. Liu *et al.* [17] proposed an ammonia micro sensor manufactured by the CMOS-MEMS technique. The sensitive material of the sensor was polyaniline, and its sensitivity was 0.88 mV/ppm. The sensitivity of the sensor in this work exceeds that of Liu *et al.* [17].

## 5. Conclusions

A zinc oxide nanorod ammonia microsensor integrated with a readout circuit manufactured by the CMOS-MEMS technique was successfully implemented. The sensitive film of the ammonia sensor prepared by the hydrothermal method was zinc oxide nanorods with porous structure, so the sensor had a fast response time. The post-process employed a wet etching to etch the sacrificial layer exposing the polysilicon electrodes, and then zinc oxide was coated on the polysilicon electrodes. The ammonia sensor resistance changed upon adsorbing ammonia gas. The readout circuit converted the resistance variation of the sensor into the voltage output. Experimental results showed that the ammonia sensor had a sensitivity of about 1.5 mV/ppm at room temperature.
